# Exosomal IRF1-loaded rat adipose-derived stem cell sheet contributes to wound healing in the diabetic foot ulcers

**DOI:** 10.1186/s10020-023-00617-6

**Published:** 2023-04-25

**Authors:** Min Wu, Jun Tu, Jinjun Huang, Huicai Wen, Yuanlin Zeng, Yingjie Lu

**Affiliations:** 1 Department of Orthopedics, Jiangxi Provincial Children’s Hospital, Nanchang, 330006 P. R. China; 2 Department of Plastic, Medical Center of Burn Plastic and Wound Repair, The First Affiliated Hospital of Nanchang University, No. 17, Yongwaizheng Street, Nanchang, 330006 Jiangxi Province P. R. China; 3 Department of Burn Surgery, Medical Center of Burn Plastic and Wound Repair, The First Affiliated Hospital of Nanchang University, Nanchang, 330006 P. R. China

**Keywords:** Diabetes mellitus, Wound healing, Fibroblasts, IRF1, miR-16-5p, SP5, Exosomes, Adipose-derived stem cell sheet

## Abstract

**Background:**

Cell-based therapy has been recognized as a novel technique for the management of diabetic foot ulcers, and cell-sheet engineering leads to improved efficacy in cell transplantation. This study aims to explore the possible molecular mechanism of the rat adipose-derived stem cell (ASC) sheet loaded with exosomal interferon regulatory factor 1 (IRF1) in foot wound healing.

**Methods:**

Rats were rendered diabetic with streptozotocin, followed by measurement of miR-16-5p expression in wound tissues. Relationship between IRF1, microRNA (miR)-16-5p, and trans-acting transcription factor 5 (SP5) was analyzed using luciferase activity, RNA pull-down, and chromatin immunoprecipitation assays. IRF1 was overexpressed in rat ASCs (rASCs) or loaded onto the rASC sheet, and then exosomes were extracted from rASCs. Accordingly, we assessed the effects of IRF1-exosome or IRF1-rASC sheet on the proliferation and migration of the fibroblasts along with endothelial cell angiogenesis.

**Results:**

miR-16-5p was poorly expressed in the wound tissues of diabetic rats. Overexpression of miR-16-5p promoted fibroblast proliferation and migration as well as endothelial cell angiogenesis, thus expediting wound healing. IRF1 was an upstream transcription factor that could bind to the miR-16-5p promoter and increase its expression. In addition, SP5 was a downstream target gene of miR-16-5p. IRF1-exosome from rASCs or the IRF1-rASC sheet facilitated the foot wound healing in diabetic rats through miR-16-5p-dependent inhibition of SP5.

**Conclusion:**

The present study demonstrates that exosomal IRF1-loaded rASC sheet regulates miR-16-5p/SP5 axis to facilitate wound healing in diabetic rats, which aids in development of stem cell-based therapeutic strategies for diabetic foot wounds.

**Supplementary Information:**

The online version contains supplementary material available at 10.1186/s10020-023-00617-6.

## Introduction

The global prevalence of diabetes mellitus (DM) is estimated to occur in over 415 million people and may double by 2040 according to the World Health Organization (Schacter and Leslie 2017). DM is a heterogeneous group of metabolic disturbances characterized by chronic hyperglycaemia (Petersmann et al. 2019), associated with a wide range of macrovascular and microvascular complications including cardiovascular disorders, diabetic retinopathy (Cole and Florez 2020). Diabetic foot ulcers are chronic wound that represents a frequent complication of DM (Ghotaslou et al. 2018). Diabetic foot ulcers lead to frequent recurrence and correlate with high mortality. Early diagnosis and effective management of diabetic foot ulcers are necessary so as to reduce the morbidity and mortality (Schmidt and Holmes 2018).

Of increasing interest is the therapeutic use of exosomes released from adipose-derived stem cells (ASCs) in facilitating wound healing in diabetic mice (Shi et al. 2020). It should be noted that allogenic transplantation of ASC sheets combined with artificial skin has been confirmed to enhance the diabetic wound healing in a rat model of type 2 DM (Kato et al. 2017). Evidence has been presented demonstrating the presence of interferon regulatory factor 1 (IRF1) in adipose-derived mesenchymal progenitors (Friesen et al. 2017). Intriguingly, IRF1 drives the proliferative, migratory, and angiogenic potential of the human umbilical vein endothelial cells (HUVECs), contributing to wound healing of diabetic foot ulcers (Cai et al. 2019). The preliminary analysis of miRGen database predicted the presence of putative binding sites between IRF1 and microRNA (miR)-16-5p. Recent evidence suggests that miR-16 modulates insulin resistance and other pathogenic conditions leading to the development of DM (Kaur et al. 2020). Additionally, forced expression of miR-16-5p shuttled by exosomes from human urine-derived stem cells can protect human podocytes from high glucose induced podocytic apoptosis (Duan et al. 2021). Moreover, starBase database analysis predicted trans-acting transcription factor 5 (SP5) as a downstream target gene of miR-16-5p. Inhibition of SP5 expression confers the promoting effect of ASC-extracellular vesicle-secreted miR-486-5p on the wound healing process (Lu et al. 2020). Hence, the current study aims to provide experimental evidence to examine the hypothesis that miR-16-5p-mediated SP5 expression was involved in IRF1 overexpression-modified exosomes (IRF1-exosome) loaded on the rat ASC (rASC) sheet in wound healing in diabetic conditions.

## Materials and methods

### Ethics statement

The current study was approved by the Animal Ethics Committee of The First Affiliated Hospital of Nanchang University Ethics Committee on Medical Research (No. 2021-028) and performed according to the Guide for the Care and Use of Laboratory Animals.

### Microarray-based profiling

DM foot ulcer-related miRNA dataset GSE68185 and ulcer skin wound healing-related miRNA dataset GSE174661 were downloaded from the Gene Expression Omnibus (GEO) database. The Kyoto encyclopedia of genes and genomes (KEGG) signaling pathways involved in miRNAs were predicted through the miRPathDB database, with the select evidence set as Experiment (union), showing the pathways involved in at least two miRNAs and miRNAs involved in at least 2 signaling pathways. miR-16-5p expression was extracted from the ulcer skin tissues (control, n = 5) and skin wound healing tissues (treat, n = 10) included in the expression profile of GSE174661, and its differential expression was evaluated using Weltch t' test statistical method with a *p* value < 0.05 deem as statistically significant. Through the miRBase database, BLAST alignment of miRNA sequences between humans, mice, and rats was conducted. The upstream transcription factors of miRNA and binding sites were predicted using miRGen database. miRmap database was used to predict binding sites of miRNA to the downstream target genes.

### DM rat model establishment

One hundred and twelve 12-week-old male Sprague–Dawley (SD) rats (weighing 185–240 g; Hunan SJA Laboratory Animal Co., Ltd., Changsha, China) were housed at 24 ± 2 °C and 50 ± 10% humidity under a 12-h light/dark cycle. The rats were acclimatized for one week before model construction. Streptozotocin (STZ; Beyotime, Shanghai, China) was dissolved in 0.1 M phosphate-citrate buffer and individually injected into the peritoneum of SD rats at a dose of 55 mg/kg. After two weeks, blood samples were harvested from the tail vein of SD rats after fasting overnight, followed by measurement of glucose levels. Glucose levels over 250 mg/mL indicated the success of DM modeling (Yang et al. 2019). The process of in vivo animal experiment is shown in Additional file 1: Fig. S1.

Normal foot tissues and foot wound tissues were isolated from mice not treated with STZ as normal control (n = 8) and non-DM control (n = 8), respectively. DM rats were given a high-fat and high-glucose diet and grouped into: DM, control, agomir negative control (NC), miR-16-5p agomir, overexpression (oe)-NC, oe-IRF1, oe-IRF1 + antagomir NC, oe-IRF1 + miR-16-5p antagomir, exosomes, IRF1-exosome, rASC sheet, and IRF1-rASC sheet groups (n = 8) (Additional file 3: Table S1).

### Construction of foot wounds in DM rats

The back of the rat foot was shaved where a 5 mm wound was created under isoflurane anesthesia. Using a 1 mL syringe, the rats were injected with 0.5 mL 50 nM agomir NC, miR-16-5p agomir, miR-16-5p antagomir, and 0.5 mL phosphate buffered saline (PBS), exosomes (1 × 10^11^ /mL), or oe-IRF1-exosome (1 × 10^11^ mouse/mL) along the wound edge and center, once a day for two days. Twenty-four hours after the second injection, the surrounding skin and the soft tissue below the wound were sutured with a 4–0 suture to prevent the wound from contraction and intervention under isoflurane anesthesia. After surgery, wounds were photographed at a fixed distance using an Olympus SP-800 UZ camera mounted on a tripod. The rats were later resuscitated and observed for their wounds daily (Liu et al. 2019). Rats were treated with agomir NC (miR4N0000001-4-5, RiboBio, Guangzhou, China), miR-16-5p agomir (miR40000785-4-5, RiboBio), antagomir NC (miR3N0000001-4-5, RiboBio), and miR-16-5p antagomir (miR30000785-4-5, RiboBio).

### Isolation and identification of rASCs

The rASCs were isolated from inguinal adipose tissues from a 12-week-old normal healthy male SD rat (weighing 250 g). The isolated adipose tissues were treated with 0.1% Type I Collagenase (Thermo Fisher Scientific, Waltham, MA) for 1 h at 37 °C. The stromal vascular part was collected after centrifugation at 700* g* for 5 min. Cells in the stromal vascular part were resuspended in α-minimum essential medium (MEM) Glutamax containing 20% fetal bovine serum (FBS) and 1% penicillin/streptomycin (Beyotime). The cells (1 × 10^6^ cells/mL) were seeded in 60 cm^2^ Primaria tissue culture dishes and cultured in a 5% CO_2_ incubator at 37 °C. After 24 h, debris was washed with PBS and fresh medium was added. Cells were transferred to a new culture dish containing 0.2% trypsin-ethylenediaminetetraacetic acid (EDTA) on day 3. Subcultures were seeded (1.7 × 10^3^ cells/cm^2^) every 3 days until passage 3.

The rASCs were characterized by detecting surface markers using flow cytometry on a CyAn ADP analyzer (Beckman Coulter., Chaska, MN) (Kato et al. 2015). The cells were probed with fluorescein isothiocyanate (FITC)-labeled CD14 (PA5-78,957, Thermo Fisher Scientific), CD19 (JF099-9, Thermo Fisher Scientific), CD105 (OTI8A1, Abcam Inc, Cambridge, UK), CD34 (EP373Y, Abcam), CD44 (EPR18668, Abcam), CD45 (ab10558, Abcam), CD73 (4G6E3, Abcam), and CD90 (ab225, Abcam) as well as major histocompatibility complex, class II, DR (HLA-DR) (1:100; BioLegend, San Diego, CA), with FITC-immunoglobulin G (IgG) (A0556, Beyotime) set as isotype control.

The rASC differentiation was further assessed. For osteogenic differentiation induction, rASCs were cultured in osteoblastic medium (Cyagen, Guangzhou, China), with the medium renewed every 2–3 days. After 14–28 days, the rASCs were stained with alizarin red (Beyotime). For adipogenic differentiation induction, rASCs were cultured in adipogenic differentiation medium (Cygen). After 3 days, the medium was replaced with maintenance medium and then with induction medium one day later. After 3 cycles of induction/maintenance medium swapping, the rASCs were cultured in the maintenance medium for another 7 days, and then the lipid accumulation in the cells was observed with oil red O staining (Beyotime). For chondrogenic differentiation induction, the suspension of rASCs (volume: 500 μL; cell density: 5 × 10^5^/mL) was centrifuged at 1200 rpm/min for 5 min. After 48 h, the rASCs became spheroids and then were cultured in the chondrogenic medium (Cyagen), with the medium renewed every 2–3 days. After 21 days, the cell granules were stained with Alcian blue (Beyotime) (Zhang et al. 2016). Images were captured using a microscope (IX71; Olympus, Tokyo, Japan).

### Isolation and identification of rASC-exosomes

The rASCs were incubated with the supernatant from lentivirus expressing IRF1 and 5 μg/mL polycoagulant (Beyotime) for 24 h. After 48 h of infection, puromycin dihydrochloride (Thermo Fisher Scientific) was used to screen the infected rASCs.

The rASCs were cultured with FBS-free rASC medium. After 48 h, the conditioned medium (CM) was harvested, from which exosomes were isolated at 4 °C by differential ultracentrifugation (Wei et al. 2017). After centrifugation at 400*g* for 10 min, the CM was centrifuged at 2000*g* for 15 min. The supernatant was collected and filtered through a 0.22 m filter. The filtered CM was concentrated to 1/30 volume following centrifugation using a 100 kDa ultrafiltration tube at 4000*g*. The concentrate was then centrifuged at 100,000*g* for 70 min to remove supernatant. The pellet was resuspended in 200 μL PBS for further use.

The morphology of the isolated exosomes was observed under a JEOL 1200EX transmission electron microscope (JEOL USA., Peabody, MA). The size distribution was analyzed using the NanoSight NS300 Nanoparticle Tracing Analyzer (NTA; Malvern Instruments Ltd., Shanghai, China). Western blot analysis was conducted for determination of surface markers (CD9 [ab92726, Abcam], ALG-2 interacting protein X [Alix; ab275377, Abcam] and tumor susceptibility 101 [Tsg101; ab12501, Abcam]) (Huang et al. 2021).

### rASC sheet construction and skin wound transplantation

The isolated rASCs were seeded (1.5 × 10^5^ cells/dish) in a 35 mm temperature-responsive dish (UpCell; CellSeed, Tokyo, Japan) and cultured in complete medium for 3 days. The cells were further cultured in complete medium containing ascorbic acid (16.4 μg/mL) for 4–5 days. After the temperature was lowered to room temperature, the cells were spontaneously separated into continuous cell sheets, which were harvested from the dish with tweezers.

The constructed sheets were placed on the wound of rats as the experimental group, and both the experimental and control groups were covered with 15 × 10 mm of artificial skin (PELNAC; Smith & Nephew, Tokyo, Japan). The skin around the wound and the soft tissues below were sutured with a 4–0 suture to prevent wound contraction and interference under isoflurane anesthesia. To protect the wound, the top of the artificial skin was sutured with a 4–0 suture using a 20 × 15 mm non-adhesive dressing (Hydrosite Plus [called ALLEVYN Non-Adhesive in the US]; Smith & Nephew, Tokyo, Japan) to keep the wound moist and absorb the exudation.

### Hematoxylin and eosin (HE) and Masson staining

Rat wound tissues were fixed with methadadine solution for 48 h and with 60% ethanol for 72 h, embedded in paraffin and cut into Sects. (4-mm thick). The sections were stained with hematoxylin, and then counterstained with eosin. The morphological and structural changes of the sections were observed under an optical microscope (Huang et al. 2020).

Sections were treated with 0.5% iodine for 10 min and with 5% sodium thiosulfate for 5 min, followed by staining in Masson ponceau-acid fuchsin solution (Guduo Biotechnology Co., Ltd., Shanghai, China) for 8 min. The sections were immersed in 2% acetic acid aqueous solution, hydrolyzed with 1% phosphomolybdic acid and stained with aniline blue. Images were captured under an optical microscope (Flores-Costa et al. 2018).

### Immunohistochemical and immunofluorescence staining

The rats were anesthetized with isoflurane and euthanized with high concentration carbon dioxide (Artwohl et al. 2006). The skin was collected from all rat wounds, fixed in 4% paraformaldehyde for 12 h, soaked in 30% sucrose at 4 °C for 12 h, and cut into 14 μm-thick sections. The sections were immunostained with rabbit anti-CD31 primary antibody (ab222783, Abcam) overnight at 4 °C using immunohistochemical kit (G1215-200 T, Seville Biotechnology, Ltd, Wuhan, China). Next, the sections were counterstained with hematoxylin and observed under an optical microscope.

In terms of immunofluorescence, the sections were blocked with 1.5% goat serum, and incubated with the rabbit primary antibodies against CD31 and α-smooth muscle actin (α-SMA) (ab150301, Abcam) overnight at 4 °C. The next day, the sections were incubated with Alexa Fluor 488 goat anti-rabbit IgG (GB25303, Seville Biotechnology Limited, Wuhan, China) and Cy3-labeled goat anti-rabbit IgG (GB21303, Seville Biotechnology Limited). Images were captured using a Leica fluorescence microscope (Zeiss Observer Z1) with a digital camera (Axiocam 503 Mono).

### Culture of fibroblasts and endothelial cells

Rat fibroblasts Rat2 (CRL-1764), and endothelial cells YPEN-1 (CRL-2222™) were purchased from American Type Culture Collection (ATCC; Manassas, VA). Endothelial cells were cultured in Dulbecco's modified eagles medium (DMEM) supplemented with 10% FBS, 100 U/mL penicillin, 100 μg/mL streptomycin and 1% endothelial cell growth supplement/heparin kit (ECGS/H, C-30140, Promocell, Germany) at 37 °C in a 5% CO_2_ incubator. Fibroblasts were cultured in high-glucose DMEM with 15% FBS, 100 U/mL penicillin, and 100 μg/mL streptomycin at 37 °C.

### Cell transfection

Cells were transfected with mimic NC (miR1N0000001-1-5, RiboBio), miR-16-5p mimic (miR10000785-1-5, RiboBio), inhibitor NC (miR2N0000001-1-5, RiboBio), or miR-16-5p inhibitor (miR20000785-1-5, RiboBio) using the HiPerFect transfection reagent (QIAGEN, Valencia, CA) as instructions described.

Lentiviral overexpression vector (LV4, GenePharma, Shanghai, China) and interference vector (pGPU6/GFP, C02007, GenePharma) were used as instructions described. Lentiviral vector carrying oe-IRF1, oe-SP5, oe-NC, short hairpin RNA (sh)-IRF1, and sh-NC were purchased from Sigma-Aldrich (St Louis, MO). The virus titer was 10^9^ TU/mL. Cells were seeded in 6-well plates at a density of 2 × 10^5^ cells/well, cultured for 24 h, and infected with the above lentiviral vectors for 72 h. The medium was replaced with fresh medium containing 4 μg/mL puromycin and cells were cultured for at least 14 days. Puromycin-resistant cells were amplified in 2 μg/mL puromycin-containing medium for 9 days and then transferred to puromycin-free medium to obtain stably knockdown or overexpression cells. The sequences of sh-IRF1-#1 was 5’-GCTAGAGATGCAGATTAATTC-3’. The groups were described in Additional file 3: Table S2.

### 5-Ethynyl-2′deoxyuridine (EdU) assay

Cells to be tested were seeded in 24-well plates were cultured in medium supplemented with EdU (Beyotime; 10 µmol/L) for 2 h. Cells were fixed with PBS solution containing 4% paraformaldehyde, and incubated with PBS with 0.5% Triton X-100. Next, the cells were stained with 4′6-diamidino-2-phenylindole (DAPI) and observed under a fluorescence microscope (Olympus) in random 6–10 fields (Zhan et al. 2018).

### Scratch test and vessel-like tube formation assay

Cells (4 × 10^5^ cells/well) were seeded in 6-well plates. When the cells reached 90–100% confluence, the plate was scratched 4–5 times vertically and slowly with a sterile 10 μL pipette tip. The cells were then cultured with serum-free medium. Images were acquired under an inverted microscope (IX53, Olympus) at 0 and 24 h to observe cell migration (Zhu et al. 2019).

The vessel-like tube formation in endothelial cells were assayed in extracellular matrix (ECM) gel as previously described (Zhou et al. 2021). A suspension (200 μL) containing 80,000 endothelial cells was covered on the ECM gel (E1270, Sigma-Aldrich), which was added to 24-well plates (200 μL/well). After 8 h, tube formation was visualized using an optical microscope.

### Dual-luciferase reporter assay

Predicted target SP5 or IRF1 wild type (WT) and mutant type (MUT) sequences were inserted into the psiCHECK2 vector (HanBio Biotechnology Co., Ltd., Shanghai, China) vector. Using Lipofectamine 2000 reagent (11668030, Thermo Fisher Scientific), the mimic-NC/miR-16-5p mimic and IRF1-WT/IRF1-MUT or SP5-WT/SP5-MUT (Additional file 3: Table S3) were co-transfected into HEK293T cells (CRL-11268; ATCC) for 48 h. Luciferase activity was detected using Dual-Luciferase® Reporter Assay System (E1910, Promega), with renilla luciferase as the loading control.

### RNA pull-down assay

After IRF1 (HanBio) was expressed in HEK293T cells with pMIR vector, the cells were transfected with WT biotinylated miR-16-5p and MUT biotinylated miR-16-5p (50 nM each), respectively for 48 h. Cells were lysed with specific lysis buffer (Ambion, Austin, Texas), and 50 μL sample cell lysate was aliquoted. The residual lysate was incubated with M-280 streptavidin beads (S3762, Sigma-Aldrich) precoated with RNase-free and yeast tRNA (Sigma-Aldrich) at 4℃ for 3 h. The bound RNA was purified by Trizol, and the enrichment of IRF-1 was detected by reverse transcription polymerase chain reaction (RT-qPCR).

### Chromatin immunoprecipitation (ChIP) assay

Antibodies against RNA polymerase II (ab238146, Abcam) and IRF1 (ab230652, Abcam) and the EZ-ChIP™ kit (17-371FR, EMD Millipore, Billerica, MA) were applied for ChIP assay. Cells were fixed with formaldehyde to generate cross-linking, and then sonicated to 300–500 bp chromatin. The lysate was added to microwells and fixed with the corresponding antibodies. DNA was released from the protein-DNA complex, purified, and eluted, followed by detection with by RT-qPCR. Both Input and IgG were used to confirm whether the detected signal was derived from the binding of specific chromatin to IRF1, or RNA polymerase II proteins.

### RNA extraction and RT-qPCR

Total RNA was extracted from cells using TRIzol method, and the extracted RNA was reversely transcribed into complementary DNA using PrimeScript RT Kit (RR047A, Takara, Dalian, China) and PrimeScript miRNA RT Kit (638315, Clontech, Palo Alto). RT-qPCR was then performed using RT-qPCR Kit (Q511-02, NanJing Vazyme Biotech Co., Ltd., Nanjing, China) on the Bio-rad real-time PCR instrument CFX96, as normalized to glyceraldehyde-3-phosphate dehydrogenase (GAPDH) and U6. Primer sequences were designed and provided by Sangon (Shanghai, China), shown in Additional file 3: Table S4. The fold changes were calculated by means of relative quantification (2^−△△Ct^ method).

### Western blot assay

Total protein was extracted from cells with radioimmunoprecipitation assay (RIPA) lysis buffer containing 1% protease inhibitor and phosphorylase inhibitors (Beyotime), with concentration assessed using a bicinchoninic acid (BCA) assay kit (A53226, Thermo Fisher Scientific). After separation by sodium dodecyl sulfate–polyacrylamide gel electrophoresis (SDS-PAGE), the protein was transferred to polyvinylidene fluoride (PVDF) membranes. After being blocked with 5% bovine serum albumin (BSA), the membranes underwent overnight incubation at 4 °C with primary rabbit antibodies against SP5 (1:1000; TF8742R, Shanghai Jingfeng Biological Technology Co., Ltd., Shanghai, China), and GAPDH (1:10,000; G9545, Sigma-Aldrich; loading control). Afterwards, the membranes were incubated with HRP-labeled goat anti-rabbit secondary antibody IgG (1:2000; ab6721, Abcam) for 2 h. The immunocomplexes on the membrane were visualized using a chemiluminometer and band intensities were quantified using ImageJ 1.48 u software.

### Statistical analysis

Statistical analysis of data in this study was conducted using SPSS 21.0 statistical software. Measurement data were presented as mean ± standard deviation. Data between two groups were compared using *t*-test and those among multiple groups were compared employing one-way analysis of variance (ANOVA), followed by Tukey’s post hoc test for multiple comparisons. Repeated measures ANOVA with Tukey’s post hoc test was applied for the comparison of data at different time points. A value of *p* < 0.05 was statistically significant.

## Results

### miR-16-5p upregulation promotes fibroblast proliferation and migration as well as endothelial cell angiogenesis

**Fig. 1:**
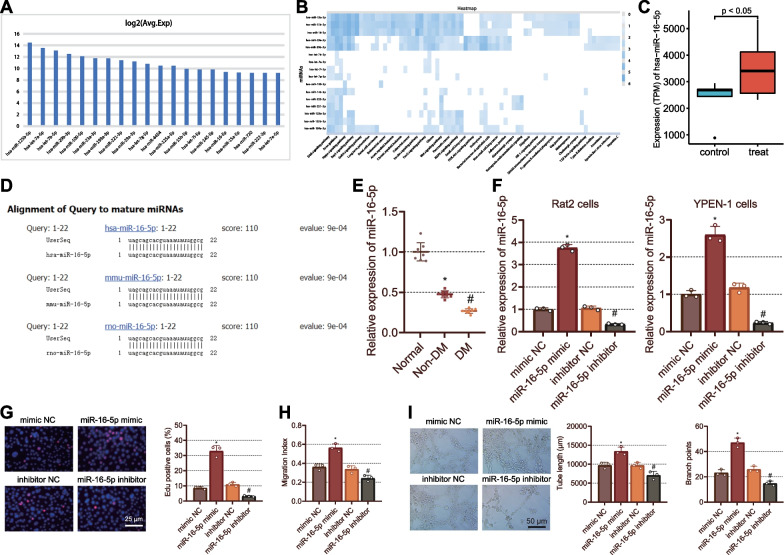
Promoting effects of miR-16-5p on fibroblast proliferation, migration, and endothelial cell angiogenesis. **A** The expression patterns of top 20 upregulated miRNAs in DM samples and non-DM samples in the GSE68185 dataset. **B** KEGG analysis of the candidate miRNAs through miRPathDB, wherein deeper color in blue refers to higher − log10 (*p* value). **C** Box plot of miR-16-5p expression in skin wound healing tissues (treat, n = 10) compared to ulcer skin tissues (control, n = 5). **D** Sequence conservation of miR-16-5p between human, mouse and rat. **E** RT-qPCR measurement of the expression of miR-16-5p in the normal foot tissues of normal rats and foot wound tissues of non-DM and DM rats. **F** Expression of miR-16-5p in Rat2 fibroblasts and YPEN-1 endothelial cells transfected with miR-16-5p mimic or miR-16-5p inhibitor. **G** The proliferation of fibroblasts transfected with miR-16-5p mimic or miR-16-5p inhibitor determined by EdU assay (red fluorescence: EdU; blue fluorescence: DAPI). **H** The Rat fibroblast migration transfected with miR-16-5p mimic or miR-16-5p inhibitor determined by scratch test. **I** Angiogenesis of YPEN-1 endothelial cells transfected with miR-16-5p mimic or miR-16-5p inhibitor detected by vessel-like tube formation assay. In panels **F**–**I**: *p < 0.05 vs. cells transfected with mimic NC. #* p* < 0.05 vs. cells transfected with inhibitor NC. Cell experiments were repeated three times. n = 8. Measurement data were presented as mean ± standard deviation. Data among multiple groups were compared by one-way ANOVA, followed by Tukey’s post hoc test for multiple comparisons

Differential analysis of the GSE68185 dataset yielded 20 significantly upregulated miRNAs in the fibroblasts of the foot (Fig. 1A). Additional KEGG functional enrichment of these miRNAs through miRPathDB database suggested close correlations of miR-16-5p with Type II DM and pathways such as Wnt pathway and insulin pathway (Fig. 1B). Additionally, statistical analysis of GSE174661 dataset suggested that miR-16-5p expression was significantly higher in skin wound healing tissues (treat, n = 10) than in ulcer skin tissues (control, n = 5) (Fig. 1C). Therefore, we speculated that miR-16-5p might regulate the wound healing rate in DM rats.

Through BLAST alignment of miRBase database, miR-16-5p was conserved in human, mouse and rat sequences (Fig. 1D). Next, a DM rat model was developed. Lower miR-16-5p expression was noted in the wounded foot tissues of non-DM rats than that in normal foot tissues of normal rats. However, miR-16-5p expression was lowered in DM rats as compared to that in non-DM rats (Fig. 1E).

Meanwhile, Rat2 fibroblasts and YPEN-1 endothelial cells were transfected with miR-16-5p mimic or inhibitor. We observed that the expression of miR-16-5p was increased in the miR-16-5p mimic-transfected cells whereas it was reduced in the miR-16-5p inhibitor-transfected cells (Fig. 1F).

In addition, a significant increase was witnessed in the percentage of EdU-positive Rat2 fibroblasts in the presence of miR-16-5p mimic, which indicated increased cell proliferation. In contrast, opposite results were noted in the presence of miR-16-5p inhibitor (Fig. 1G). The results of scratch test displayed that miR-16-5p mimic augmented the Rat2 fibroblast migration while miR-16-5p inhibitor repressed their migration (Fig. 1H). In addition, there was an increase in tube length and branches in response to miR-16-5p mimic, which was indicative of increased angiogenesis, while miR-16-5-p inhibitor led to contrasting results (Fig. 1I). The above results demonstrated that overexpression of miR-16-5p promoted the proliferative and migratory potential of fibroblasts, and angiogenic potential of endothelial cells.

### miR-16-5p upregulation facilitates foot wound healing in DM rats

**Fig. 2:**
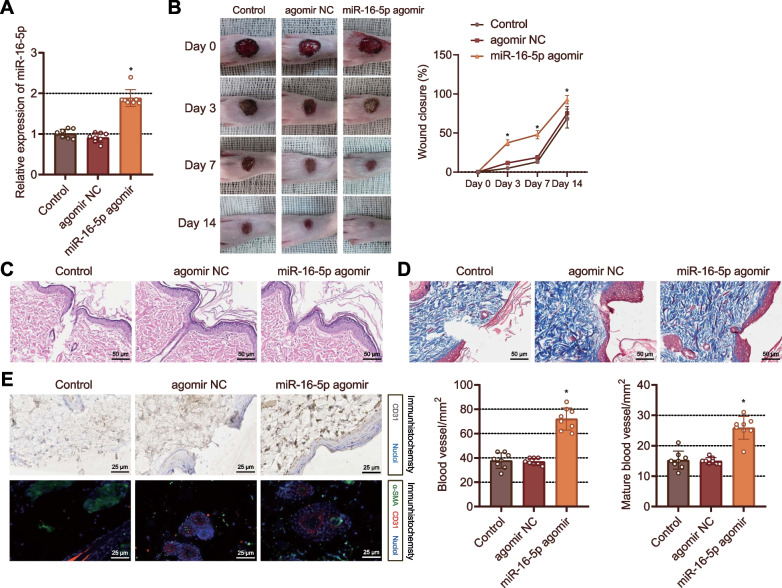
miR-16-5p overexpression induces foot wound healing in DM rats. **A** RT-qPCR detection of the miR-16-5p expression in the foot wound tissues of DM rats treated with agomir NC or miR-16-5p agomir. *p < 0.05 vs. the agomir NC-treated rats. **B** Skin wound diameter changes in DM rats treated with agomir NC or miR-16-5p agomir. * p < 0.05 vs. agomir NC-treated rats. **C** Morphological and structural changes of foot wound tissues observed by HE staining. **D** Masson staining of collagen deposition in the foot wound tissues of DM rats treated with agomir NC or miR-16-5p agomir. **E** The number of newly formed and mature vessels in foot wounds of DM rats treated with agomir NC or miR-16-5p agomir assessed by immunohistochemical and immunofluorescent staining. *p < 0.05 vs. agomir NC-treated rats. n = 8. Measurement data were presented as mean ± standard deviation. Data among multiple groups were compared by one-way ANOVA, followed by Tukey’s post hoc test for multiple comparisons. Repeated measures ANOVA with Tukey’s post hoc test was applied for the comparison of data at different time points

The effect of miR-16-5p on foot wound healing in DM rats was the next focus of this study. Elevated expression of miR-16-5p was witnessed in the wound tissues of rats treated with miR-16-5p agomir (Fig. 2A). On day 3, the wound area was reduced in the miR-16-5p agomir-treated rats. On day 7, the wound healing was initiated with callus formed in the agomir NC-treated rats, while the wound area was decreased in the miR-16-5p agomir-treated rats. On day 14, the foot wound of the miR-16-5p agomir-treated rats was largely healed without traces, and the wound traces remained in the rats treated with agomir NC (Fig. 2B).

HE staining results showed that the wounds of rats treated with miR-16-5p agomir were almost closed and the newly formed epithelium covered almost the entire cells while the newly formed epithelium covered only part of the wounds of rats treated with agomir NC (Fig. 2C). Furthermore, Masson staining results revealed increased collagen deposition and thickened wavy collagen fibers in rats injected with agomir NC or miR-16-5p agomir, with miR-16-5p agomir-treated rats showing more obvious changes (Fig. 2D). Immunohistochemistry and immunofluorescence staining results exhibited augmented CD31 and α-SMA expression in the wound tissues of the rats treated with miR-16-5p agomir than that in rats treated with agomir NC, implying dense and mature blood vessels (Fig. 2E). Based on the above results, overexpression of miR-16-5p-5p promoted angiogenesis and the subsequent foot wound healing in DM rats.

### IRF1 specifically binds to miR-16-5p and promotes its expression in fibroblasts

**Fig. 3:**
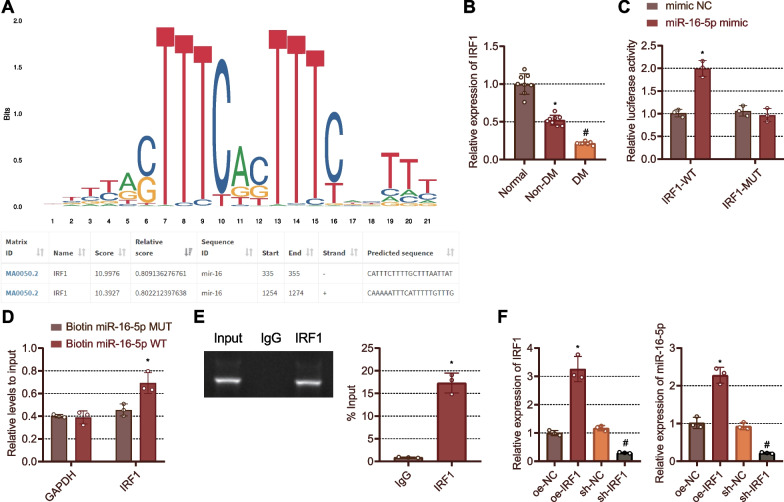
IRF1 targets miR-16-5p and increases its expression in fibroblasts. **A** The motif and binding site of the upstream transcription factor IRF1 of miR-16-5p predicted by miRGen database. **B** IRF1 expression in normal foot tissues of normal rats and foot wound tissues of non-DM and DM rats detected by RT-qPCR. *p < 0.05 vs. normal rats; #* p* < 0.05 vs. non-DM rats. **C** Binding of IRF1 to the miR-16-5p promoter identified by dual-luciferase reporter assay. * p < 0.05 vs. the pMIR group. **D** Direct binding of IRF1 to miR-16-5p detected by RNA pull-down assay. **E** Binding of IRF1 to miR-16-5p validated by ChIP assay. *p < 0.05 vs. IgG group. **F** The expression of IRF1 and miR-16-5p in fibroblasts treated with oe-IRF1 or sh-IRF1 determined by RT-qPCR. *p < 0.05 vs. the fibroblasts treated with oe-NC. #*p* < 0.05 vs. the fibroblasts treated with sh-NC. Cell experiments were repeated three times. n = 8. Measurement data were presented as mean ± standard deviation. Data among multiple groups were compared by one-way ANOVA, followed by Tukey’s post hoc test for multiple comparisons

The motif and the binding site of the upstream transcription factor IRF1 of miR-16-5p were predicted by miRGen database (Fig. 3A). Furthermore, it was witnessed that IRF1 expression was decreased in the wound tissues of non-DM rats compared to normal foot tissues of normal rats, while a more reduction was noted in the wound tissues of the DM rats (Fig. 3B). The results of luciferase activity assay suggested that miR-16-5p mimic contributed to significantly increased luciferase activity in cells co-transfected with IRF1-WT, but it failed to alter those transfected with IRF1-MUT (Fig. 3C). RNA pull-down assay results confirmed that miR-16-5p-WT enriched more IRF1 than miR-16-5p-MUT (Fig. 3D). This binding was further validated by ChIP assay (Fig. 3E). These results indicated that IRF1 could bind to miR-16-5p. In addition, the expression of IRF1 and miR-16-5p was raised in oe-IRF1-treated Rat2 fibroblasts but suppressed in the sh-IRF1-treated Rat2 fibroblasts (Fig. 3F). Overall, IRF1 was identified as an upstream transcription factor of miR-16-5p, and IRF1 could bind to the miR-16-5p and promote its expression.

### IRF1 enhances foot wound healing in DM rats by elevating the expression of miR-16-5p

**Fig. 4:**
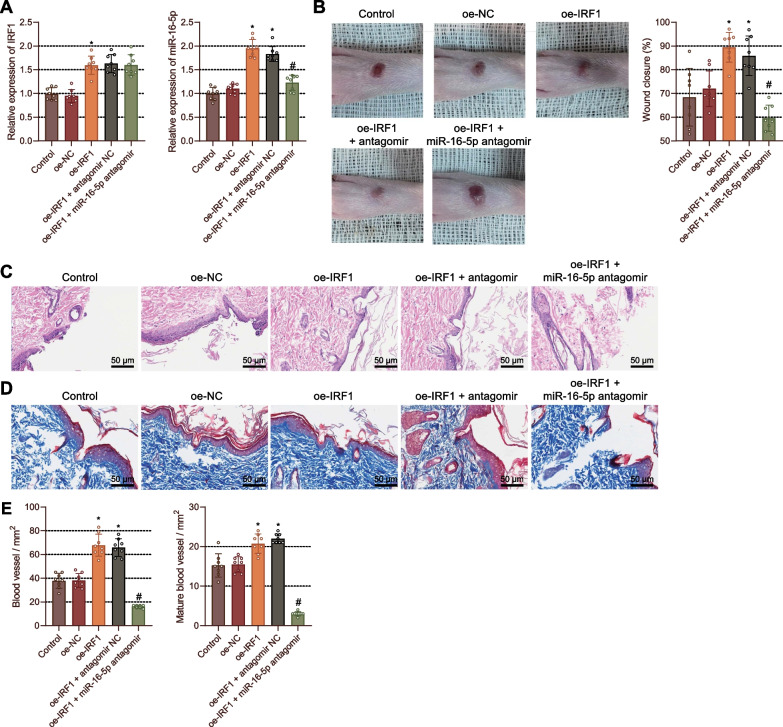
IRF1 enhances the wound healing in DM rats by upregulating the expression of miR-16-5p. DM rats were treated with oe-IRF1, oe-IRF1 + antagomir NC, or oe-IRF1 + miR-16-5p antagomir. **A** The expression of IRF1 and miR-16-5p in the rat wound tissues determined by RT-qPCR on day 14. *p < 0.05 vs. oe-NC-treated rats. #*p* < 0.05 vs. oe-IRF1 + antagomir NC-treated rats. **B**, Changes in foot wound diameter in rats on day 14. *p < 0.05 vs. oe-NC-treated rats. #*p* < 0.05 vs. oe-IRF1 + antagomir NC-treated rats. **C** Morphological and structural changes of rat wound tissues observed by HE staining. **D** Collagen deposition in the rat wound tissues analyzed by Masson staining on day 14. *p < 0.05 vs. oe-NC-treated rats. #*p* < 0.05 vs. oe-IRF1 + antagomir NC-treated rats. **E** Quantitative analysis of newly formed (left) and mature (right) blood vessels in rat wound tissues on day 14 assessed by immunohistochemical and immunofluorescent staining. *p < 0.05 vs. oe-NC-treated rats. #*p* < 0.05 vs. oe-IRF1 + antagomir NC-treated rats. n = 8. Measurement data were presented as mean ± standard deviation. Data among multiple groups were compared by one-way ANOVA, followed by Tukey’s post hoc test for multiple comparisons. Repeated measures ANOVA with Tukey’s post hoc test was applied for the comparison of data at different time points

Based on the above-mentioned results, we hypothesized and validated whether IRF1 promoted wound healing by increasing miR-16-5p expression in vivo. RT-qPCR results validated an increase in the IRF1 and miR-16-5p expression in the wound tissues of DM rats treated with oe-IRF1 or oe-IRF1 + antagomir NC on day 14. In contrast, oe-IRF1 + miR-16-5p antagomir treatment led to lower miR-16-5p expression than oe-IRF1 + antagomir NC treatment (Fig. 4A).

As shown in Fig. 4B, the wound area was decreased after treatment with oe-IRF1 or oe-IRF1 + antagomir NC on day 14. The worse wound healing was observed after treatment oe-IRF1 + miR-16-5p antagomir. The wound largely healed without traces in the rats treated with oe-IRF1 or oe-IRF1 + antagomir NC.

In addition, HE staining results showed that oe-IRF1 or oe-IRF1 + antagomir NC treatment increased the length of newly formed epithelium and enhanced wound healing, and consequently the wounds were almost closed and the newly formed epithelium covered almost the entire cells. Conversely, the wound length and the newly formed epithelium were slightly smaller in the rats treated with oe-IRF1 + miR-16-5p antagomir than in the rats treated with oe-IRF1 + antagomir NC, with a part of the wound not closed (Fig. 4C). Furthermore, Masson staining results revealed the occurrence of collagen deposition and wavy fibers in all groups on day 14. Increased collagen deposition and thickened wavy collagen fibers were observed in the presence of oe-IRF1 or oe-IRF1 + antagomir NC, while less collagen deposition and reduced wavy collagen fibers were observed in the presence of oe-IRF1 + miR-16-5p antagomir (Fig. 4D).

The results of immunohistochemistry and immunofluorescence staining suggested that on day 14, blood vessels in the rats treated with oe-IRF1 or oe-IRF1 + antagomir NC were denser and maturer than those in the control rats, while the oe-IRF1 + miR-16-5p antagomir led to suppressed formation and maturation of blood vessels relative to the oe-IRF1 + antagomir NC (Fig. 4E). These lines of results indicated that overexpression of IRF1 could contribute to the wound healing in DM rats by upregulating the expression of miR-16-5p.

### miR-16-5p targets SP5 and inhibits its expression in fibroblasts

**Fig. 5:**
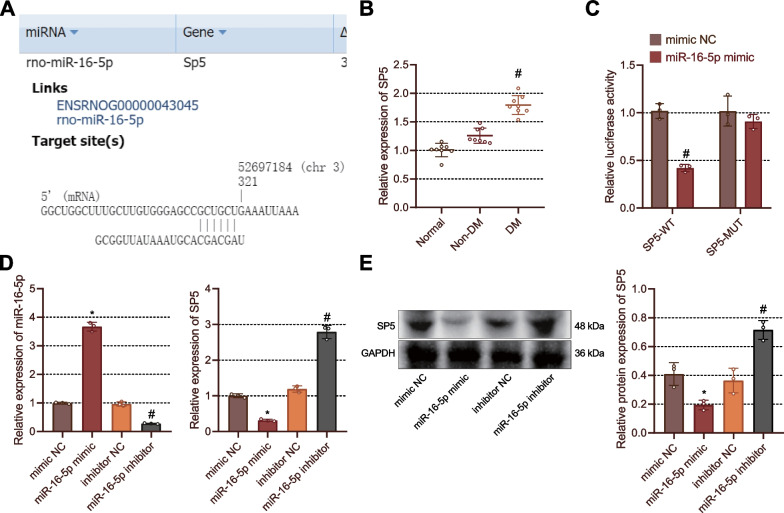
SP5 is a target gene of miR-16-5p. **A** Binding sites between miR-16-5p and SP5 predicted by the starBase database. **B** RT-qPCR detection of SP5 expression in rat foot wound tissues of non-DM and DM rats. *p < 0.05 vs. normal rats; #*p* < 0.05 vs. non-DM rats. **C** The binding of miR-16-5p to SP5 in HEK293T cells validated by dual-luciferase reporter assay. #*p* < 0.05 vs. mimic NC-transfected HEK293T cells. **D** SP5 expression in fibroblasts transfected with miR-16-5p mimic or miR-16-5p inhibitor detected by RT-qPCR. *p < 0.05 vs. fibroblasts transfected with mimic NC; # *p* < 0.05 vs. fibroblasts transfected with inhibitor NC. **E** Western blots of SP5 protein in fibroblasts transfected with miR-16-5p mimic or miR-16-5p inhibitor. *p < 0.05 *vs.* fibroblasts transfected with mimic NC. #*p* < 0.05 *vs.* fibroblasts transfected with inhibitor NC. Cell experiments were repeated three times. n = 8. Measurement data were presented as mean ± standard deviation. Data among multiple groups were compared by one-way ANOVA, followed by Tukey’s post hoc test for multiple comparisons

Next, we sought to explore the downstream mechanism of miR-16-5p in promoting wound healing. starBase database predicted SP5 as a downstream target gene of miR-16-5p evidenced by the presence of binding sites between them (Fig. 5A). Of note, SP5 was highly expressed in the foot wound tissues of DM rats (Fig. 5B). The results of dual-luciferase reporter assay demonstrated that the luciferase activity upon transfection with SP5-WT was reduced in the presence of miR-16-5p mimic, but no alteration was observed in the presence of SP5-MUT (Fig. 5C). Meanwhile, transfection with miR-16-5p mimic in Rat2 fibroblasts diminished the expression of SP5 while miR-16-5p inhibitor resulted in increased expression of SP5 (Fig. 5D, E). Taken together, miR-16-5p could bind to SP5 and inhibit its expression in fibroblasts.

### IRF1-exosome by rASCs promotes wound healing in DM rats

In this study, we first isolated rASCs from rats, identified rASCs by flow cytometry, and verified the differentiation ability of rASCs (Additional file 2: Fig. S2A, B). It was observed that rASCs were successfully isolated with typical differentiation capacity and could be used to extract exosomes. oe-NC/oe-IRF1 was transfected into rASCs, and exosomes/IRF1-exosome was isolated after induction. According to the results of TEM and NTA, the microvesicles secreted from rASCs ranged from 30 to 150 nm. Western blot analysis further verified that exosome markers CD9, Alix and Tsg101 were positively expressed in exosomes/IRF1-exosome (Additional file 2: Fig. S2C, D), which indicated that exosomes were successfully isolated.

**Fig. 6:**
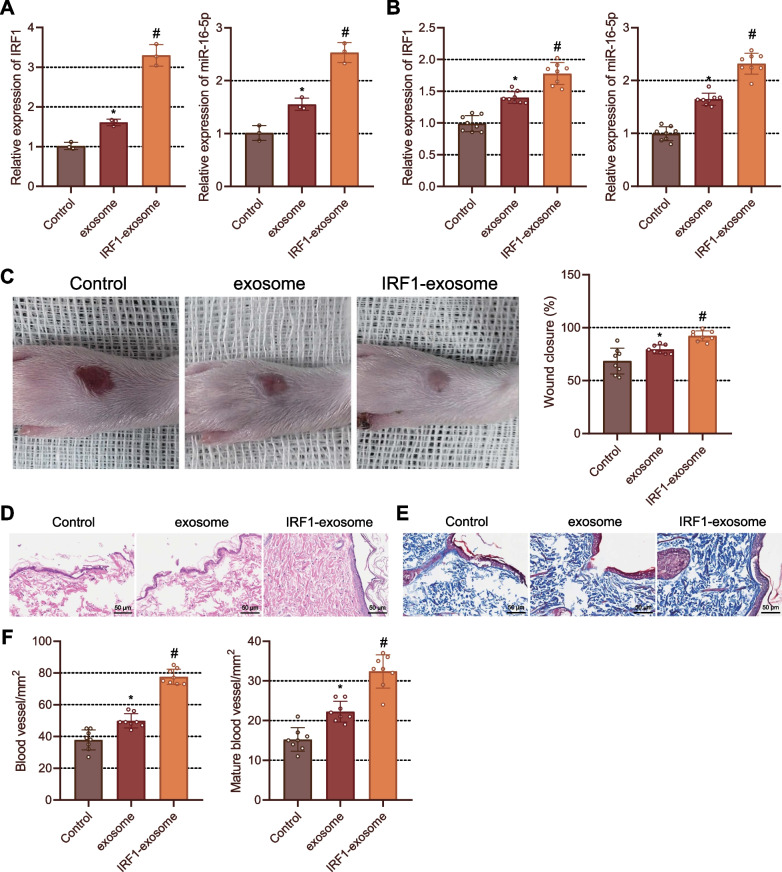
IRF1**-**exosome facilitates wound healing in DM rats. **A** The expression of IRF1 and miR-16-5p in rASCs treated with exosomes or IRF1-exosome determined by RT-qPCR. **B** The expression of IRF1 and miR-16-5p in foot wound tissues of rats on day 14 determined by RT-qPCR. **C** Wound diameter changes in rats on day 14. **D** Morphological and structural changes of rat wound tissues observed on day 14 by HE staining. **E** Collagen deposition in the rat wound tissues analyzed on day 14 by Masson staining. **F** Quantitative analysis of newly formed (left) and mature (right) blood vessels in rat wound tissues on day 14. *p < 0.05 vs. PBS-treated DM rats. #*p* < 0.05 vs. exosome-treated rats. Cell experiments were repeated three times. n = 8. Measurement data were presented as mean ± standard deviation. Data among multiple groups were compared by one-way ANOVA, followed by Tukey’s post hoc test for multiple comparisons

Moreover, upregulation in the expression of IRF1 and miR-16-5p was noted in rASCs treated with exosomes compared with that in control rASCs, and elevation in IRF1 and miR-16-5p expression was observed in rASCs treated with IRF1-exosome than in those treated with exosomes (Fig. 6A). In vivo data exhibited that on day 14, IRF1 and miR-16-5p expression increased in the foot wound tissues of DM rats treated with exosomes compared with PBS-treated DM rats while higher IRF1 and miR-16-5p expression was observed in the DM rats treated with IRF1-exosome than in DM rats treated with exosomes (Fig. 6B). On day 14, wounds were almost healed in all rats, and better wound healing was found after treatment with IRF1-exosome and exosomes (Fig. 6C).

On day 14, HE staining data showed the wound area was not significantly reduced but the new epithelial formation was enhanced in the exosome-treated DM rats than in the PBS-treated DM rats. The wounds were almost closed upon treatment with IRF1-exosome, and the new epithelium covered almost the entire cells (Fig. 6D). Additionally, the results of Masson staining indicated increased collagen deposition and thickened wavy collagen fibers in the DM rats treated with exosomes compared to PBS-treated DM rats on day 14; however, treatment with IRF1-exosome led to more collagen deposition than treatment with exosomes, and the arrangement of wavy collagen fibers was similar to normal skin (Fig. 6E).

Furthermore, immunohistochemistry and immunofluorescence staining results suggested that exosome treatment led to dense and mature blood vessels in wound tissues, which were more obvious following treatment with IRF1-exosome on day 14 (Fig. 6F). The above results indicated that IRF1-exosome could induce miR-16-5p expression to promote wound healing in DM rats.

### IRF1-exosome stimulates fibroblast proliferation and migration and endothelial cell angiogenesis by facilitating the miR-16-5p-mediated inhibition of SP5

**Fig. 7:**
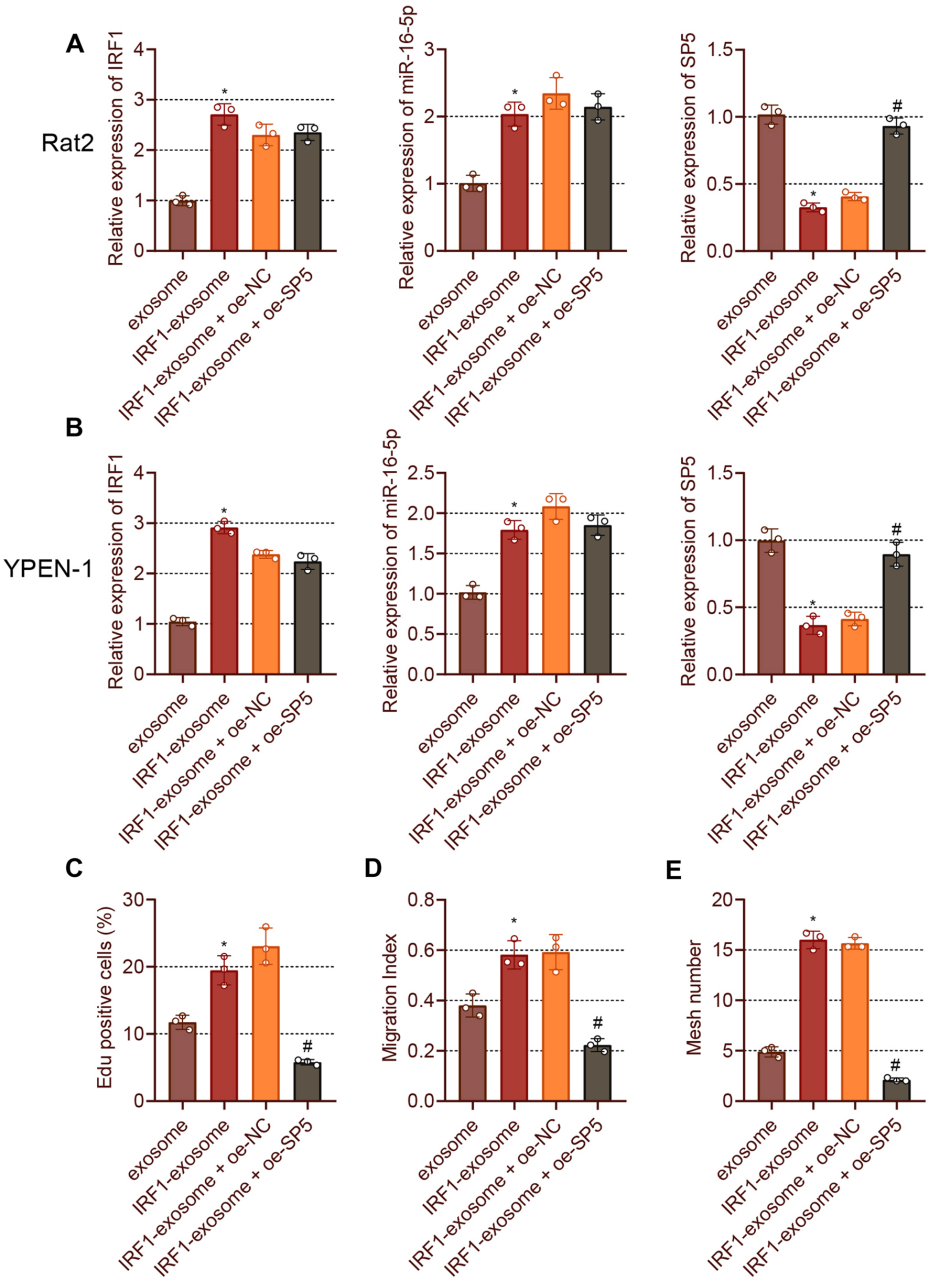
IRF1-exosome triggers fibroblast proliferation and migration, and endothelial cell angiogenesis via the miR-16-5p/SP5 axis. Fibroblasts or endothelial cells were treated with exosomes, IRF1-exosome or IRF1-exosome + oe-SP5. **A** and **B**, IRF1, miR-16-5p and SP5 expression in Rat2 fibroblasts (**A**) and YPEN-1 endothelial cells (**B**) measured by RT-qPCR. **C** Rat2 fibroblast proliferation determined by EdU. **D** Rat2 fibroblast migration determined by scratch test. **E** YPEN-1 endothelial cell angiogenesis as detected by vessel-like tube formation assay. *p < 0.05 vs. exosome-treated fibroblasts or endothelial cells. # *p* < 0.05 vs. IRF1-exosome + oe-NC-treated fibroblasts or endothelial cells. Cell experiments were repeated three times. Measurement data were presented as mean ± standard deviation. Data among multiple groups were compared by one-way ANOVA, followed by Tukey’s post hoc test for multiple comparisons

We then aimed to explore the molecular mechanism by which IRF1 regulates the miR-16-5p/SP5 axis and affects foot wound healing in DM rats. According to RT-qPCR data, higher IRF1 and miR-16-5p expression and lower SP5 expression were noted in IRF1-exosome-treated Rat2 fibroblasts and YPEN-1 endothelial cells than those in exosome-treated cells. Besides, treatment with oe-SP5 induced an increase in the SP5 expression but failed to affect IRF1 and miR-16-5p expression in the IRF1-exosome-treated Rat2 fibroblasts and YPEN-1 endothelial cells (Fig. 7A, B).

In addition, Rat2 fibroblast proliferation and migration were found to be increased upon treatment with IRF1-exosome, the effects of which were reversed after restoration of SP5 expression (Fig. 7C, D). The angiogenesis of endothelial cells was noted to be enhanced following treatment with IRF1-exosome while SP5 re-expression reversed this enhancement (Fig. 7E). Cumulatively, exosome-oe-IRF1 could induce miR-16-5p expression and downregulate SP5 expression, thus promoting fibroblast proliferation and migration as well as endothelial cell angiogenesis.

### IRF1-rASC sheet induces foot wound healing in DM rats

**Fig. 8:**
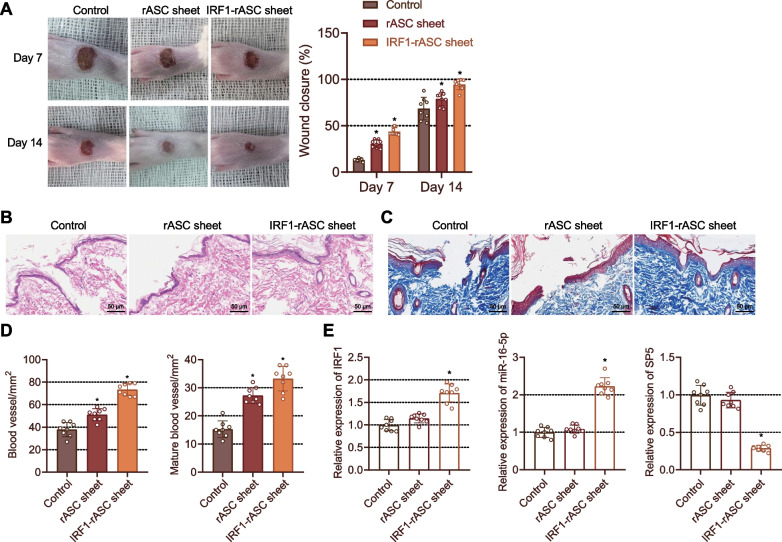
IRF1-exosome loaded into the rASC sheet promotes wound healing in DM rats. DM rats were implanted with rASC sheet or IRF1-rASC sheet at the wound. **A** Wound healing rate of rats on day 7 and day 14. *p < 0.05 vs. control rats at the same timepoints. **B** Morphological and structural changes of rat wound tissues observed on day 14 by HE staining. **C** Collagen deposition in the rat wound tissues analyzed on day 14 by Masson staining. **D** Quantitative analysis of newly formed (left) and mature (right) blood vessels in rat wound tissues on day 14. *p < 0.05 vs. control rats. **E** Expression of IRF1, miR-16-5p and SP5 in rat wound tissues detected on day 14 by RT-qPCR. *p < 0.05 vs. control rats. n = 8. Data among multiple groups were compared by one-way ANOVA, followed by Tukey’s post hoc test for multiple comparisons. Repeated measures ANOVA with Tukey’s post hoc test was applied for the comparison of data at different time points

We constructed rASC sheet and IRF1-rASC sheet and transplanted them to the foot wound of DM rats, and covered with artificial skin. On day 7, treatment with the rASC sheet accelerated wound healing, while IRF1-rASC sheet led to much faster wound healing. On day 14, the wounds in the rats treated with the IRF1-rASC sheet were almost completely closed, but those in the rats treated with the rASC sheet were not fully healed than in the control rats, accompanied with an increase in the newly formed epithelium (Fig. 8A).

On day 14, HE staining results showed that the formation of new epithelium and wound healing were enhanced upon treatment with IRF1-rASC sheet. Besides, the wounds of rats treated with IRF1-rASC sheet were almost closed and the newly formed epithelium covered the entire wound (Fig. 8B). Masson staining results suggested increased collagen deposition in the wound tissues of rats treated with the IRF1-rASC sheet than in those treated with the rASC sheet or control rats, with the arrangement of wavy collagen fibers similar to normal skin on day 14 (Fig. 8C).

Immunohistochemistry and immunofluorescence staining results suggested that the blood vessels were dense and mature in the rats treated with IRF1-rASC sheet on day 14 (Fig. 8D). RT-qPCR data revealed that on day 14, the expression of IRF1 and miR-16-5p was augmented while that of SP5 was reduced in wound tissues of rats treated with the IRF1-rASC sheet (Fig. 8E). The aforementioned results suggested that IRF1 loaded into the rASC sheet could induce the expression of miR-16-5p and then promote wound healing in DM rats.

## Discussion

The key findings of the present study revealed that the IRF1-exosome loaded into the rASC sheet could induce miR-16-5p expression and downregulate the expression of SP5, resulting in the promotion of fibroblast proliferation and migration and endothelial cell angiogenesis, whereby facilitating the foot wound healing in DM rats.

We found that miR-16-5p was poorly expressed in the foot wound tissues of DM rats, while overexpression of miR-16-5p conferred improvement of wound healing of DM rats. miR-16-5p expression has been documented to be decreased in type 1 DM patients (Gao and Zhao 2020). Despite the lack of investigations on the role of miR-16-5p in wound healing and DM, a recent study has suggested that exosomal miR-16-5p originating from human urine-derived stem cells can attenuate high glucose-induced apoptosis of human podocytes (Duan et al. 2021). Recent evidence has also documented that miR-16-5p in induced pluripotent stem cell-derived micro-vesicles can increase re-epithelization and collagen deposition in the wounds, thus accelerating deep second-degree burn wound healing (Yan et al. 2020), which is in line with our finding. Evidence exists suggesting that miR-16-5p was highly expressed in the serum of patients with gestational DM, which may be a potential diagnostic biomarker of gestational DM (Cao et al. 2017; Juchnicka et al. 2022). In addition, it was documented that miR-16-5p was poorly expressed in the serum of patients with type 1 diabetic nephropathy, which indicated that miR-16-5p might participate in the pathogenesis of type 1 diabetic nephropathy (Assmann et al. 2019). In the present study, we unraveled that miR-16-5p was poorly expressed in the skin of DM patients. Therefore, miR-16-5p may play different physiological roles in different types of diabetes and its complications.

Fibroblasts exert critical roles in the repairing processes, from the late inflammatory phase to the final epithelization of injured tissues; their proliferation and migration are capable of stimulating wound healing processes in the damaged skin (Addis et al. 2020). Intriguingly, miR-16-5p was reported to enhance keratinocyte migration but failed to affect the proliferation of the cells (Jiang et al. 2021). In this study, overexpression of miR-16-5p was found to promote fibroblast proliferation and migration, and endothelial cell angiogenesis, which support the promoting impact of miR-16-5p on foot wound healing in DM rats. Prior evidence has suggested that the typical wound repair includes four stages: (1) platelet mediated hemostasis; (2) inflammation; (3) scar formation caused by proliferation and migration of keratinocytes to the wound for re-epithelialization, fibroblast migration, contraction and collagen deposition; (4) wound vessel resolution and scar tissue remodeling (Cash and Martin 2016). Scar formation is the final result of abnormal wound healing (Jiang et al. 2021).

IRF1 has been reported to bind to the promoter of several miRs and activates their expression, such as miR-134 and miR-29b (Ma et al. 2020; Yuan et al. 2015). This study first revealed that IRF1 specifically bound to miR-16-5p and promoted its expression in fibroblasts, thus enhancing the foot wound healing in DM rats. IRF1 can promote the proliferative, migratory, and angiogenic capacity of the HUVECs, resulting in augmented wound healing in diabetic foot ulcers (Cai et al. 2019). In addition, ASC-exosomes can accelerate the proliferation and migration of fibroblasts and angiogenesis of HUVECs (Ma et al. 2022). Meanwhile, exosomes secreted by ASCs can enhance the proliferation and angiopoiesis in endothelial progenitor cells in a high glucose environment and promote wound healing (Li et al. 2018). The current study demonstrated that exosomes from the rASCs overexpressing IRF1 could accelerate wound healing in DM rats, which was associated with the miR-16-5p-mediated inhibition of SP5. Furthermore, our in vivo data demonstrated the beneficial role of IRF1-loaded rASC sheet in the wound healing. It should be noted that other regulatory factors, such as lncRNA SNHG16 and circ-CUX1 which have been reported as miR-16-5p regulators, might modulate the expression of miR-16–5 in wound healing in the diabetic foot ulcers. Future studies are needed to examine other possible mechanisms. Further exploration of the current work identified that miR-16-5p bound to the 3’UTR of SP5 mRNA and could negatively regulate its expression. Inhibiting the expression of SP5 facilitates proliferation and migration of human skin fibroblast and angiogenesis of human microvascular endothelial cells, thus promoting the wound healing process (Lu et al. 2020). These findings collectively suggest that miR-16-5p-mediated inhibition of SP5 engaged in the promotion of foot wound healing in DM rats. This study further provided both in vitro and in vivo evidence that miR-16-5p-mediated inhibition of SP5 may be the mechanism underpinning the promoting role of IRF1-loaded rASC sheet or IRF1-loaded rASC-derived exosomes in wound healing.

## Conclusions

**Fig. 9:**
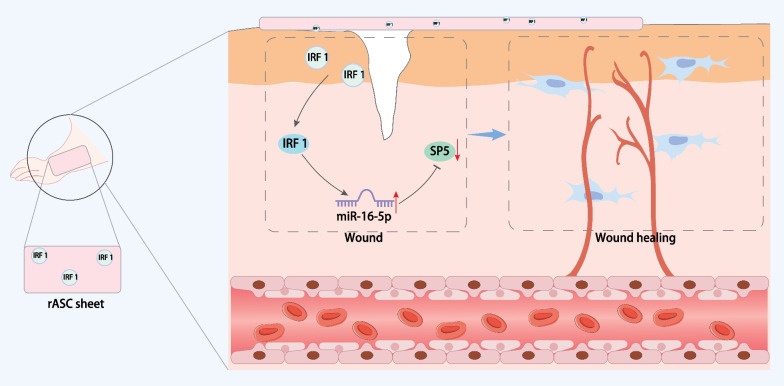
Schematic representation of the molecular mechanisms whereby IRF1-exosome loaded into the rASC sheet affects foot wound healing in DM rats. IRF1 loaded into the rASC sheet can induce miR-16-5p expression and target SP5, consequently promoting fibroblast proliferation and migration and endothelial cell angiogenesis and accelerating foot wound healing in DM rats

In conclusion, IRF1 loaded into the rASC sheet can augment the proliferation and migration of fibroblasts, and endothelial cell angiogenesis, promoting the foot wound healing in DM rats (Fig. 9). Exosomes from rASC overexpressing IRF1 or IRF1-loaded rASC sheet presents a fresh perspective for the development of future therapeutic approaches against DM-related disorders. However, there are still several limitations in the present study: (1) We only studied the DM model induced by streptozotocin, and did not use other models of wound healing for in-depth exploration; (2) Our study preliminarily showed that IRF1 overexpression-modified exosomes induced miR-16-5p expression to target and inhibit SP5, thereby promoting the healing of foot skin wounds in DM rats. However, the related molecular changes in this mechanism need to be further confirmed in diabetes foot ulcer patients; (3) The wound healing rate in the presence of oe-IRF1 + miR-16-5p antagonist was much lower than that upon oe-NC, indicating that transcription factors other than IRF-1 may regulate the expression of miR-16-5p, the regulatory mechanism of which needs to be further explored in future research.

## Supplementary Information

**Additional file 1: Figure S1.** Flow chart of in vivo animal experiments.

**Additional file 2: Figure S2.** Isolation and identification of rASCs and exosomes. A, rASCs markers were detected by flow cytometry. B, Alizarin red staining to detect osteogenic differentiation ability of rASCs, oil red O staining to detect the fat differentiation ability of rASCs, and Alcian blue staining to detect the cartilage differentiation ability of rASCs. C, The morphology of exosomes observed by TEM. D, Western blot was used to detect exosomes and IRF1 exosomes.

**Additional file 3: Table S1.** Rat grouping. **Table S2.** Cell grouping **Table S3.** SP5-WT/SP5-MUT sequences. **Table S4.** Primer sequences for RT-qPCR.

## Data Availability

The data that supports the findings of this study are available on request from the corresponding author upon reasonable request.

## Abbreviations

DM: Diabetes mellitus
ASCs: Adipose-derived stem cells
IRF1: Interferon regulatory factor 1
HUVECs: Human umbilical vein endothelial cells
miR: MicroRNA
IRF1-exosome: IRF1 overexpression-modified exosomes
SP5: Trans-acting transcription factor 5
rASC: Rat ASC
GEO: Gene Expression Omnibus
SD: Sprague-Dawley
NC: Negative control
oe: Overexpression
PBS: Phosphate buffered saline
α-MEM: α-Minimum essential medium
FBS: Fetal bovine serum
EDTA: Ethylenediaminetetraacetic acid
FITC: Fluorescein isothiocyanate
HLA-DR: Major histocompatibility complex, class II, DR
IgG: Immunoglobulin G
CM: Conditioned medium
Alix: ALG-2 interacting protein X
Tsg101: Tumor susceptibility 101
HE: Hematoxylin and eosin
α-SMA: α-Smooth muscle actin
DMEM: Dulbecco's modified eagles medium
sh: Short hairpin RNA
EdU: 5-Ethynyl-2′deoxyuridine
DAPI: 4′6-Diamidino-2-phenylindole
WT: Wild type
MUT: Mutant type
RT-qPCR: Reverse transcription polymerase chain reaction
ChIP: Chromatin immunoprecipitation
GAPDH: Glyceraldehyde-3-phosphate dehydrogenase
RIPA: Radioimmunoprecipitation assay
BCA: Bicinchoninic acid
SDS-PAGE: Sodium dodecyl sulfate–polyacrylamide gel electrophoresis
PVDF: Polyvinylidene fluoride
BSA: Bovine serum albumin
KEGG: Kyoto encyclopedia of genes and genomes
ANOVA: Analysis of variance (ANOVA)

## References

[CR1] Addis R, Cruciani S, Santaniello S, Bellu E, Sarais G, Ventura C (2020). Fibroblast proliferation and migration in wound healing by phytochemicals: evidence for a novel synergic outcome. Int J Med Sci.

[CR2] Artwohl J, Brown P, Corning B, Stein S, Force AT (2006). Report of the ACLAM task force on rodent euthanasia. J Am Assoc Lab Anim Sci.

[CR3] Assmann TS, Recamonde-Mendoza M, Costa AR, Punales M, Tschiedel B, Canani LH (2019). Circulating miRNAs in diabetic kidney disease: case-control study and in silico analyses. Acta Diabetol.

[CR4] Cai HA, Huang L, Zheng LJ, Fu K, Wang J, Hu FD (2019). Ginsenoside (Rg-1) promoted the wound closure of diabetic foot ulcer through iNOS elevation via miR-23a/IRF-1 axis. Life Sci.

[CR5] Cao YL, Jia YJ, Xing BH, Shi DD, Dong XJ (2017). Plasma microRNA-16-5p, -17-5p and -20a-5p: novel diagnostic biomarkers for gestational diabetes mellitus. J Obstet Gynaecol Res.

[CR6] Cash JL, Martin P (2016). Myeloid cells in cutaneous wound repair. Microbiol Spectr.

[CR7] Cole JB, Florez JC (2020). Genetics of diabetes mellitus and diabetes complications. Nat Rev Nephrol.

[CR8] Duan YR, Chen BP, Chen F, Yang SX, Zhu CY, Ma YL (2021). Exosomal microRNA-16-5p from human urine-derived stem cells ameliorates diabetic nephropathy through protection of podocyte. J Cell Mol Med.

[CR9] Flores-Costa R, Alcaraz-Quiles J, Titos E, Lopez-Vicario C, Casulleras M, Duran-Guell M (2018). The soluble guanylate cyclase stimulator IW-1973 prevents inflammation and fibrosis in experimental non-alcoholic steatohepatitis. Br J Pharmacol.

[CR10] Friesen M, Camahort R, Lee YK, Xia F, Gerszten RE, Rhee EP (2017). Activation of IRF1 in human adipocytes leads to phenotypes associated with metabolic disease. Stem Cell Rep.

[CR11] Gao X, Zhao S (2020). miRNA-16-5p inhibits the apoptosis of high glucose-induced pancreatic beta cells via targeting of CXCL10: potential biomarkers in type 1 diabetes mellitus. Endokrynol Pol.

[CR12] Ghotaslou R, Memar MY, Alizadeh N (2018). Classification, microbiology and treatment of diabetic foot infections. J Wound Care.

[CR13] Huang X, Liang P, Jiang B, Zhang P, Yu W, Duan M (2020). Hyperbaric oxygen potentiates diabetic wound healing by promoting fibroblast cell proliferation and endothelial cell angiogenesis. Life Sci.

[CR14] Huang J, Yu M, Yin W, Liang B, Li A, Li J (2021). Development of a novel RNAi therapy: engineered miR-31 exosomes promoted the healing of diabetic wounds. Bioact Mater.

[CR15] Jiang Y, Xiang C, Zhong F, Zhang Y, Wang L, Zhao Y (2021). Histone H3K27 methyltransferase EZH2 and demethylase JMJD3 regulate hepatic stellate cells activation and liver fibrosis. Theranostics.

[CR16] Juchnicka I, Kuzmicki M, Niemira M, Bielska A, Sidorkiewicz I, Zbucka-Kretowska M (2022). miRNAs as predictive factors in early diagnosis of gestational diabetes mellitus. Front Endocrinol (lausanne).

[CR17] Kato Y, Iwata T, Morikawa S, Yamato M, Okano T, Uchigata Y (2015). Allogeneic transplantation of an adipose-derived stem cell sheet combined with artificial skin accelerates wound healing in a rat wound model of type 2 diabetes and obesity. Diabetes.

[CR18] Kato Y, Iwata T, Washio K, Yoshida T, Kuroda H, Morikawa S, et al. Creation and transplantation of an adipose-derived stem cell (ASC) Sheet in a diabetic wound-healing model. J Vis Exp. 2017;(126). 10.3791/54539 PMC5614015 28809824

[CR19] Kaur P, Kotru S, Singh S, Behera BS, Munshi A (2020). Role of miRNAs in the pathogenesis of T2DM, insulin secretion, insulin resistance, and beta cell dysfunction: the story so far. J Physiol Biochem.

[CR20] Li X, Xie X, Lian W, Shi R, Han S, Zhang H (2018). Exosomes from adipose-derived stem cells overexpressing Nrf2 accelerate cutaneous wound healing by promoting vascularization in a diabetic foot ulcer rat model. Exp Mol Med.

[CR21] Liu X, Wang Y, Zhang X, Zhang X, Guo J, Zhou J (2019). MicroRNA-296-5p promotes healing of diabetic wound by targeting sodium-glucose transporter 2 (SGLT2). Diabetes Metab Res Rev.

[CR22] Lu Y, Wen H, Huang J, Liao P, Liao H, Tu J (2020). Extracellular vesicle-enclosed miR-486-5p mediates wound healing with adipose-derived stem cells by promoting angiogenesis. J Cell Mol Med.

[CR23] Ma Z, Li K, Chen P, Pan Q, Li X, Zhao G (2020). MiR-134, mediated by IRF1, suppresses tumorigenesis and progression by targeting VEGFA and MYCN in osteosarcoma. Anticancer Agents Med Chem.

[CR24] Ma J, Zhang Z, Wang Y, Shen H (2022). Investigation of miR-126-3p loaded on adipose stem cell-derived exosomes for wound healing of full-thickness skin defects. Exp Dermatol.

[CR25] Petersmann A, Muller-Wieland D, Muller UA, Landgraf R, Nauck M, Freckmann G (2019). Definition, classification and diagnosis of diabetes mellitus. Exp Clin Endocrinol Diabetes.

[CR26] Schacter GI, Leslie WD (2017). Diabetes and bone disease. Endocrinol Metab Clin North Am.

[CR27] Schmidt BM, Holmes CM (2018). Updates on diabetic foot and charcot osteopathic arthropathy. Curr Diab Rep.

[CR28] Shi R, Jin Y, Hu W, Lian W, Cao C, Han S (2020). Exosomes derived from mmu_circ_0000250-modified adipose-derived mesenchymal stem cells promote wound healing in diabetic mice by inducing miR-128-3p/SIRT1-mediated autophagy. Am J Physiol Cell Physiol.

[CR29] Wei F, Ma C, Zhou T, Dong X, Luo Q, Geng L (2017). Exosomes derived from gemcitabine-resistant cells transfer malignant phenotypic traits via delivery of miRNA-222-3p. Mol Cancer.

[CR30] Yan Y, Wu R, Bo Y, Zhang M, Chen Y, Wang X (2020). Induced pluripotent stem cells-derived microvesicles accelerate deep second-degree burn wound healing in mice through miR-16-5p-mediated promotion of keratinocytes migration. Theranostics.

[CR31] Yang X, Cao Z, Wu P, Li Z (2019). Effect and mechanism of the bruton tyrosine kinase (Btk) inhibitor ibrutinib on rat model of diabetic foot ulcers. Med Sci Monit.

[CR32] Yuan L, Zhou C, Lu Y, Hong M, Zhang Z, Zhang Z (2015). IFN-gamma-mediated IRF1/miR-29b feedback loop suppresses colorectal cancer cell growth and metastasis by repressing IGF1. Cancer Lett.

[CR33] Zhan Y, Chen Z, Li Y, He A, He S, Gong Y (2018). Long non-coding RNA DANCR promotes malignant phenotypes of bladder cancer cells by modulating the miR-149/MSI2 axis as a ceRNA. J Exp Clin Cancer Res.

[CR34] Zhang H, Yu N, Zhou Y, Ma H, Wang J, Ma X (2016). Construction and characterization of osteogenic and vascular endothelial cell sheets from rat adipose-derived mesenchymal stem cells. Tissue Cell.

[CR35] Zhou C, Wei W, Ma J, Yang Y, Liang L, Zhang Y (2021). Cancer-secreted exosomal miR-1468-5p promotes tumor immune escape via the immunosuppressive reprogramming of lymphatic vessels. Mol Ther.

[CR36] Zhu J, Liu B, Wang Z, Wang D, Ni H, Zhang L (2019). Exosomes from nicotine-stimulated macrophages accelerate atherosclerosis through miR-21-3p/PTEN-mediated VSMC migration and proliferation. Theranostics.

