# Supplementary material for: Exosomal IRF1-loaded rat adipose-derived stem cell sheet contributes to wound healing in the diabetic foot ulcers
Source: Mol Med. 2023 Apr 25;29:60. doi: 10.1186/s10020-023-00617-6 (PMC10131451; doi:10.1186/s10020-023-00617-6)
Supplement: Supplementary file 3 — Additional file 3: Table S1. Rat grouping. Table S2. Cell grouping Table S3. SP5-WT/SP5-MUT sequences. Table S4. Primer sequences for RT-qPCR. [file 10020_2023_617_MOESM3_ESM.docx]

**Table S1** Rat grouping

| Group | Interventions |
| --- | --- |
| normal | normal foot tissues of normal rats |
| non-DM | foot wound tissues of normal rats |
| DM | foot wound tissues of DM rats |
| Control | foot wound tissues of DM rats treated with PBS |
| agomir NC | foot wound tissues of DM rats treated with agomir NC |
| miR-16-5p agomir | foot wound tissues of DM rats treated with miR-16-5p agomir |
| oe-NC | foot wound tissues of DM rats treated with oe-NC |
| oe-IRF1 | foot wound tissues of DM rats treated with oe-IRF1 |
| oe-IRF1 + antagomir NC | foot wound tissues of DM rats treated with both oe-IRF1 and antagomir NC |
| oe-IRF1 + miR-16-5p antagomir | foot wound tissues of DM rats treated with both oe-IRF1 and miR-16-5p antagomir |
| exosome | foot wound tissues of DM rats treated with exosome |
| IRF1-exosome | foot wound tissues of DM rats treated with IRF1-exosome |
| rASC sheet | foot wound tissues of DM rats treated with rASC sheet |
| IRF1-rASC sheet | foot wound tissues of DM rats treated with IRF1-rASC sheet |

Note: DM, diabetes mellitus; NC, negative control; PBS, phosphate buffer saline; miR, microRNA; oe, overexpression; IRF1, interferon regulatory factor 1; rASC, rat adipose-derived stem cell.

**Table S2** Cell grouping

| Group | Interventions |
| --- | --- |
| mimic NC | cells transfected with mimic NC |
| miR-16-5p mimic | cells transfected with miR-16-5p mimic |
| inhibitor NC | cells transfected with inhibitor NC |
| miR-16-5p inhibitor | cells transfected with miR-16-5p inhibitor |
| oe-NC | cells transfected with oe-NC plasmid |
| sh-NC | cells transduced with lentivirus carrying sh-NC plasmid |
| oe-IRF1 | cells transduced with lentivirus carrying oe-IRF1 plasmid |
| sh-IRF1 | cells transduced with lentivirus carrying sh-IRF1 plasmid |
| oe-SP5 | cells transduced with lentivirus carrying oe-SP5 plasmid |
| exosome | cells treated with exosomes |
| IRF1-exosome | cells treated with exosomes from rASCs-overexpressing IRF1 |
| IRF1-exosome + oe-NC | cells treated with IRF1-exosome and oe-NC plasmid |
| IRF1-exosome + oe-SP5 | cells treated with IRF1-exosome and oe-SP5 plasmid |

Note: NC, negative control; miR, microRNA; oe, overexpression; sh, short hairpin RNA; IRF1, interferon regulatory factor 1.

**Table S3** SP5-WT/SP5-MUT sequences

| Item | Sequence |
| --- | --- |
| SP5-WT | 5’-TTGCTTGTGGGAGCCGCTGCTG-3’ |
| SP5-MUT | 5’-TTGCTTGTGGGAGCCCGACGAG-3’ |

Note: WT, wild type; MUT, mutant type.

**Table S4** Primer sequences for RT-qPCR

| Gene | Sequence |
| --- | --- |
| IRF1 (rat) | Forward: 5’-GTACAACTTGCAGGTGTCGC-3’  Reverse: 5’-GCTGCCACTCAGACTGTTCA-3’ |
| miR-16-5p (rat) | Forward: 5’-TAGCAGCACGTAAATATTGGCG-3’  Reverse: Reverse Universal Sequence |
| SP5 (rat) | Forward: 5’-TCATGCGAAGCGACCACCT-3′  Reverse: 5’-CAATGGTGCTGGGACGGATG-3′ |
| U6 (rat) | Forward: 5’-CTCGCTTCGGCAGCACA -3′  Reverse: Reverse Universal Sequence |
| GAPDH (rat) | Forward: 5’-CGCTAACATCAAATGGGGTG-3′  Reverse: 5’-TTGCTGACAATCTTGAGGGAG-3′ |

Note: RT-qPCR, reverse transcription-quantitative polymerase chain reaction; IRF1, interferon regulatory factor 1; miR, microRNA; TGF-β1, transforming growth factor beta 1; GAPDH, glyceraldehyde-3-phosphate dehydrogenase.
